# Spatial variation and determinants of childhood anemia among children aged 6 to 59 months in Ethiopia: further analysis of Ethiopian demographic and health survey 2016

**DOI:** 10.1186/s12887-021-02901-y

**Published:** 2021-11-09

**Authors:** Tiruneh Ayele Jember, Destaw Fetene Teshome, Lemma Derseh Gezie, Chilot Desta Agegnehu

**Affiliations:** 1 Department of Epidemiology and Biostatistics, Institute of Public Health, College of Medicine and Health Sciences, University of Gondar, Gondar, Ethiopia; 2 School of Nursing, College of Medicine and Health Sciences and Comprehensive specialized hospital, University of Gondar, Gondar, Ethiopia

**Keywords:** Childhood, Anemia, Spatial, Distribution

## Abstract

**Background:**

The magnitude of childhood anemia was increased from time to time. Thus, Even if the Ethiopian government applied tremendous efforts, anemia in children continues as a major public health problem. There is limited evidence on the spatial variation of and determinant factors of childhood anemia at the national level. Therefore, this study aimed to explore spatial distribution and determinants of anemia among children aged 6 to 59 months in Ethiopia.

**Method:**

A stratified two-stage cluster sampling technique was used in Ethiopian Demographic Health Survey 2016 data. In this study 8602 children aged 6–59 months were included. Bernoulli model was used to explore the presence of purely spatial clusters of Anemia in children in age 6–59 months using Sat scan. ArcGIS version 10.3 was used to know the distribution of anemia cases across the country. A mixed-effects Logistic regression model was used to identify determinant factors of anemia.

**Results:**

The finding indicates that the spatial distribution of childhood anemia was non-random in the country with Moran’s I: 0.65, *p* < 0.001. The SaT scan analysis identified a total of 180 significant primary clusters located in the Somali and Afar regions (LLR = 14.47, *P*-value< 0.001, RR = 1.47). Age of child 12–23 months (AOR = 0, 68, 95%CI: 0.55, 0.85), 24–35 months (AOR = 0.38, 95%CI: 0.31, 0.47), and36–47 months (AOR = 0.25, 95%CI, 0.20, 0.31), working mother (AOR = 0.87, 95%CI: 0.76, 0.99), anemic mother (AOR = 1.53, 95%CI, 1.35, 1.73), had fever in the last 2 weeks (AOR = **1**.36,95%CI:1.13, 1.65), moderate stunting (AOR = 1.31,95%CI: 1.13, 1.50),Severely stunting (AOR = 1.82,95%CI: 1.54, 2.16), religion, wealth index, and number of under-five children in the household were statistically significant associated with childhood anemia.

**Conclusion:**

Spatial variation of childhood anemia across the country was non-random. Age of the child, wealth index, stunting, religion, number of under-five children in the household, fever in the last 2 weeks, anemic mother, and working status of the mother were determinants of childhood anemia. Therefore, interventions should be a priority concern for high-risk (hot spot) areas regarding allocation of resources and improved access to health facilities, and to reduce the consequence of anemia among the generation policymakers and concerned bodies should be implemented these specific determinant factors.

## Background

Anemia is a disease that is characterized by a decreased number of red blood cells or hemoglobin levels that result in the insufficient oxygen-carrying capacity of blood to meet the cellular metabolic demand of the body [1]. Iron deficiency is the major cause of anemia and there are also other causes of anemia like, nutritional deficiency, acute and chronic inflammations that affect the synthesis of hemoglobin and production [1, 2].

Globally, anemia affects more than 27% of the world’s population which is nearly 1.93 billion. However, there is a high burden of anemia cases in low and middle-income countries which is more than 89% [3]. Among the global burden of anemia two-third of under-five/ preschool children in Africa and southeast Asia were anemic [4]. On the other hand, according to World Health Organization (WHO) 2017 report, the global prevalence of anemia in under-five children was 41.7% whereas in the Africa region was 59% [5]. In sub-Saharan Africa including Ethiopia, the national prevalence of anemia among under five or preschool children was above 40% [6–8]. For example, in Ethiopia, the prevalence of anemia was persistently increased among 6–59 months of children from 44% in 2011 to 56% in 2016 within 5 years [6, 7].

World Health Organization defined as anemia is a major public health problem when prevalence is above 40% and Therefore, anemia is a worldwide public health problem that affects both developing and developed countries [4]. It increased mortality and morbidity of human health as well as poor social and economic development [4]. It occurs in all population groups of a human beings. However, children and pregnant women were the more risk age groups for anemia. The result of anemia in children is very severe and complicated like increasing child mortality, impaired cognitive and physical development [2, 4]. Previous various factors were associated with childhood anemia. For example, household wealth index [9–13], Family size [14–16], giving more diversified diet [12, 17, 18], stunting [7–10, 12, 14, 19], malaria infection [10, 11, 20], anemic mothers [13, 16, 19] history of diarrhea [12, 21], and history of fever [11, 13, 18] were determinant factors of childhood anemia.

Even though the Ethiopian government and different partners applied tremendous efforts to control the disease, anemia in under 5 years of children continues as a major public health problem [18]. As you know still anemia is a high burden of disease among under-five children and it varies from country to country, region to region. Identifying geographical variations of anemia among children is very important to prioritize and design targeted prevention and intervention programs to reduce the burden of anemia among children at the national level. Therefore, understanding the spatial variation and determinants of anemia among this group of children is important to design effective interventions and to manage program resources fairly.

**Fig. 1:**
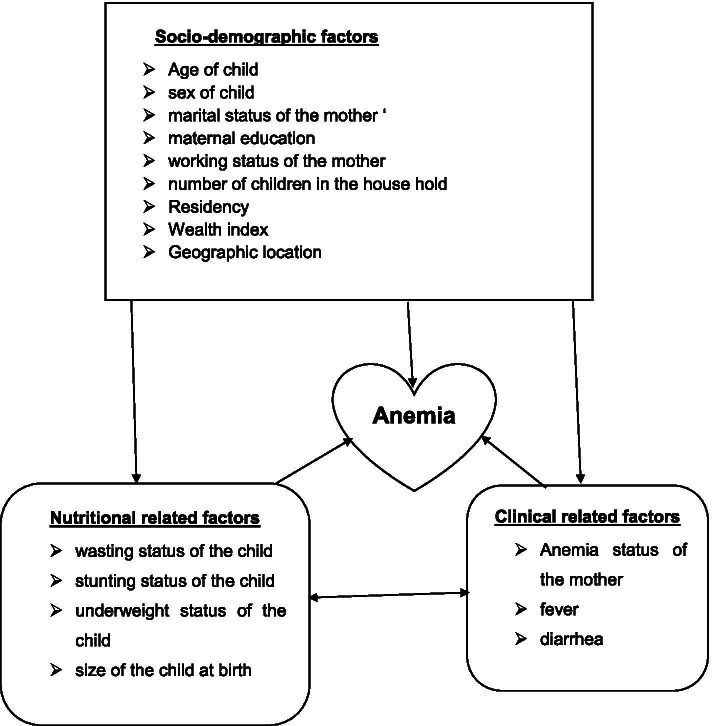
Conceptual framework of the study adopted from kinds of literature [9–25]

Identifying and handling factors of childhood anemia is very important to reduce the prevalence of childhood anemia and allocate resources fairy and give more emphasis for regions that had a high distribution of childhood anemia for a better outcome. Thus, this study is designed to assess spatial distribution and determinants of anemia among children aged 6 to 59 months in Ethiopia. The conceptual frame work is described in detail (Fig. 1).

## Method

### Study area and data source

**Fig. 2:**
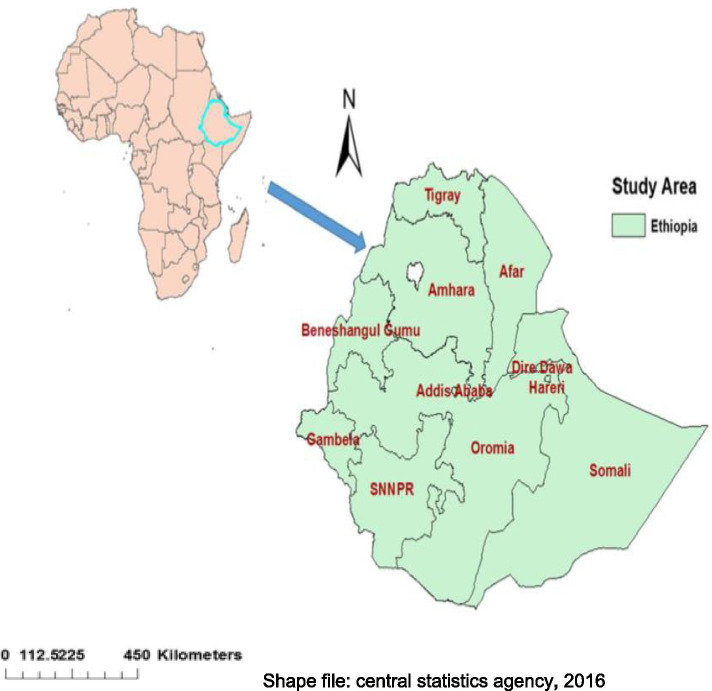
Map of Study Area

The Ethiopian Demographic and Health Survey (EDHS) is a community-based cross-sectional study conducted from 18 January to 27 June 2016. The study was conducted in Ethiopia (3^o^-14^o^ N and 33^o^ - 48°E), situated at the eastern horn of Africa (Fig. 2). The country covers 1.1 million square kilometers and has a great geographical diversity, which ranges 4550 m above sea level down to the Afar depression to 110 m below sea level [26]. There are nine regional states and two city administrations subdivided into 68 zones, 817 districts, and 16,253 kebeles (lowest local administrative units of the country in the administrative structure of the country) [7]. The source of the data for this study was the Ethiopian Demographic and Health Survey (EDHS) 2016 (*N* = 7794) and used to assess the spatial variation and determinants of childhood anemia among children age 6–59 months in Ethiopia.

### Sample size determination and sampling procedure

EDHS used a two-stage cluster sampling technique. Since Ethiopia has 9 regional states and 2 administrative cities. Administratively, regions in Ethiopia are divided into zones, and zones, into administrative units called woreda. Each woreda is further subdivided into the lowest administrative unit, called kebeles. During the 2007 census, each kebele was subdivided into census enumeration areas (EA), which were convenient for the implementation of the census [7].

A stratified two-stage cluster sampling procedure was employed where EA is the sampling unit for the first stage and households for the second stage. In 2016 EDHS, a total of 645 EAs (202 in urban areas and 443 in rural areas) were selected with probability proportional to EA size (based on the 2007 housing and population census) and with independent selection in each sampling stratum. Of this 18,008 households were included. A total of 8602 children were interviewed. But in the present study, a total of 7794 children the age of 6–59 months were included in the analysis. The source population was all births from reproductive-age women within 5 years before the survey in Ethiopia and all births from reproductive-age women in the selected enumeration areas within 5 years before the survey were the study population. Birth’s from reproductive age women within 5 years before the survey within enumeration areas with missed global positioning system (GPS) cells were excluded for spatial analysis.

### Outcome variable

The outcome variable for this study was anemia, which was dichotomized as anemic and not anemic. Individuals are considered to be not anemic was defined as Adjusted concentration of blood hemoglobin greater than or equal to 11 mg/dl and those individuals with less than 11 mg/dl were anemic [1]. Anemia status was determined based on hemoglobin concentration in blood adjusted to the altitude.

### Independent variables

The independent variables were classified as: socio-demographic factors, nutritional factors, clinical factors, and service-related factors.

The socio-demographic factors were the sex of a child, age of child, residence, educational status of the mother, maternal age, husband’s educational status, the religion of mother, wealth index, working status of the mother, and a number of children in the household. The nutritional factors were stunting status of a child, wasting status of child, and size of child at birth. The clinical factors were also maternal anemic status, diarrhea in a child in the last 2 weeks, fever in a child in the last 2 weeks, and cough in a child in the last 2 weeks and the service-related factors were taking of vitamin A in the last 6 months, taking of iron pills or sprinkles or syrup and taking of drugs for intestinal parasites in the last 6 months.

### Data management and statistical analysis

Descriptive and summary statistics were done using STATA version 14 after extraction and edition of data from EDHS 2016 child data set. Since EDHS data had hierarchical and clustering nature, the assumption of independence among observations was violated. This implies a need to consider the between-cluster variability by using advanced models. The goodness of fit test was checked using Intraclass correlation (ICC) and deviance [27]. So logistic regression (non-anemic child = 0, anemic child = 1), and GLMM (generalized linear mixed model) were fitted. Then the GLMM was selected based on the result of Akaikie Information Criteria (AIC) and Bayesian information criteria (BIC). The model with the smallest AIC value was chosen. Variables having a *p*-value up to 0.2 in the bi-variable analysis were selected to fit the model in the multi-variable analysis. Finally, a p-value less than 0.05 in the multivariable model of mixed-effects logistic regression was used to select variables that had a statistically significant association with anemia.

### Spatial autocorrelation

ArcGIS version 10.3 was used for Moran’s I analysis. The Global Moran’s I spatial statistic measures were used to measure spatial autocorrelation by taking the total data set and producing a single output value that ranges from − 1 to+ 1. Global Moran’s I value closes to − 1 which indicates dispersed childhood anemia, whereas Moran’s I value closest to + 1 indicted clustered childhood anemia and the Moran’s I value is 0 which indicates randomly distributed childhood anemia. Moran’s I (*P*-value < 0.05) indicates the presence of spatial autocorrelation.

### Hot spot analysis (Getis-OrdGi* statistic)

Getis-OrdGi* statistics were computed to measure how spatial autocorrelation varies over the study location by calculating GI* statistics for each area. Z-score is computed to determine the statistical significance of clustering, and the *p*-value is computed for the significance [28]. Statistical output with high GI* indicates “hotspot” whereas low GI* means a “cold spot [29–31].

### Spatial interpolation

It is very difficult and expensive in terms of resources and time to collect reliable data in all areas of the country to know the burden of a certain event. Therefore, part of a certain area can be predicted by using observed data using a method called interpolation. The spatial interpolation technique is used to predict childhood anemia in the un-sampled areas in the country based on sampled EAs. There are different deterministic and geostatistical interpolation methods. Among those methods, ordinary Kriging and empirical Bayesian Kriging are considered the best method since it incorporates the spatial autocorrelation and it statistically optimizes the weight [32]. The ordinary Kriging spatial interpolation method was used for this study for predictions of childhood anemia in unobserved areas of Ethiopia.

### Disease cluster detection and spatial scan statistical analysis

Spatial scan statistical analysis was employed to test for the presence of statistically significant spatial hotspots/clusters of childhood anemia using Kuldorff’s SaT Scan version 9.6 software. The spatial scan statistic uses a scanning window that moves across the study area. Children with anemia were taken as cases and without it as controls to fit the Bernoulli model. The numbers of cases in each location had Bernoulli distribution and the model required data for cases, controls, and geographic coordinates. The default maximum spatial cluster size of < 50% of the population was used, as an upper limit, which allowed both small and large clusters to be detected and ignored clusters that contained more than the maximum limit.

For each potential cluster, a likelihood ratio test statistic and *p*-value were used to determine if the number of observed childhood anemia within the potential cluster was significantly higher than expected or not. The primary and secondary clusters are identified and assigned *p*-values and ranked based on their likelihood ratio test, based on 999 Monte Carlo replications.

## Result

### Socio-demographic characteristics

The EDHS of 2016 includes 8602 children aged 6 to 59 months in the survey. Among these children, 7815(90.8%) were granted the consent statement for hemoglobin. Of the total of 7815 children whose consent statement is granted, the hemoglobin level was determined for 7794 children which gives a 90.6% response rate of the study. Their mean age was found to be 32 months (SD ± 0.18 months). Almost half, 48.6%, of the children were females. The Majority of study participants, 83%, had rural residency. Regarding the educational status of their mothers, two-third (65%) of mothers had no formal education (Table 1).

**Table 1:** A Socio-demographic characteristic of the children aged 6 to 59 months and their parents in Ethiopia from January 18 to June 27 in 2016 (*N* = 7794)

Variable	Categories	Frequency	Percent
**Age of the child in months**	6–11	914	11.7
	12–23	1765	22.7
	24–35	1702	21.8
	36–47	1656	21.3
	48–59	1757	22.5
**Sex of the child**	Male	4010	51.4
	Female	3784	48.6
**Residence**	Urban	1327	17.0
	Rural	6467	83.0
**Religion of mothers**	Orthodox	2350	30.2
	Protestant	1417	18.2
	Muslim	3837	49.2
	Others	190	2.4
**Maternal educational level**	No education	5084	65.2
	Primary	1981	25.4
	Secondary	486	6.3
	Higher	243	3.1
**Maternal age in years**	15–24	1757	22.5
	25–29	2355	30.2
	30–34	1778	22.8
	35–48	1904	24.5
**Wealth index of child’s family**	Poorest	2854	36.6
	Poorer	1387	17.8
	Middle	1156	14.8
	Richer	983	12.6
	Richest	1414	18.2
**Region**	Tigray	828	10.6
	Afar	744	9.6
	Amhara	783	10.0
	Oromia	1220	15.7
	Somali	1019	13.0
	Benishangul	659	8.5
	SNNPR	995	12.8
	Gambella	509	6.5
	Harari	373	4.8
	Addis Ababa	305	3.9
	Dire Dawa	359	4.6

### Spatial distribution of childhood anemia in Ethiopia

**Fig. 3:**
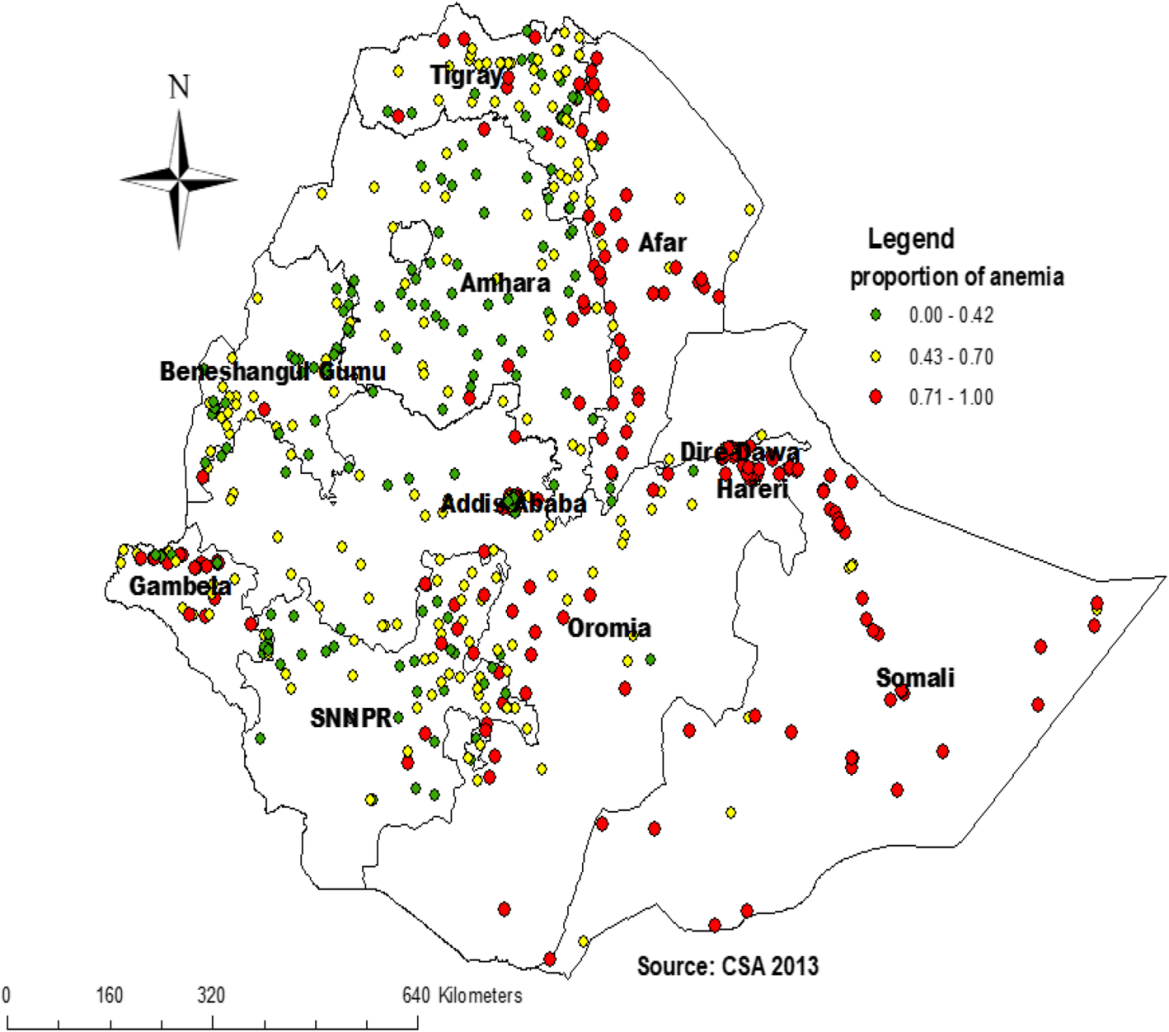
Spatial distribution of childhood anemia in Ethiopia, 2016

A total of 622 clusters were considered for spatial analysis of childhood anemia. The red color indicates a high proportion of childhood anemia whereas the green color indicates a low proportion of childhood anemia. A high proportion of childhood anemia occurred in southwest Somali, the eastern and southern part of Oromia, south Afar, Hareri Northern part of Gambella, and Dire Dawa whereas a low proportion of childhood anemia were aggregated in Amhara, Addis Ababa, west of SNNP, and east of Benshangul (Fig. 3).

### Spatial autocorrelation

**Fig. 4:**
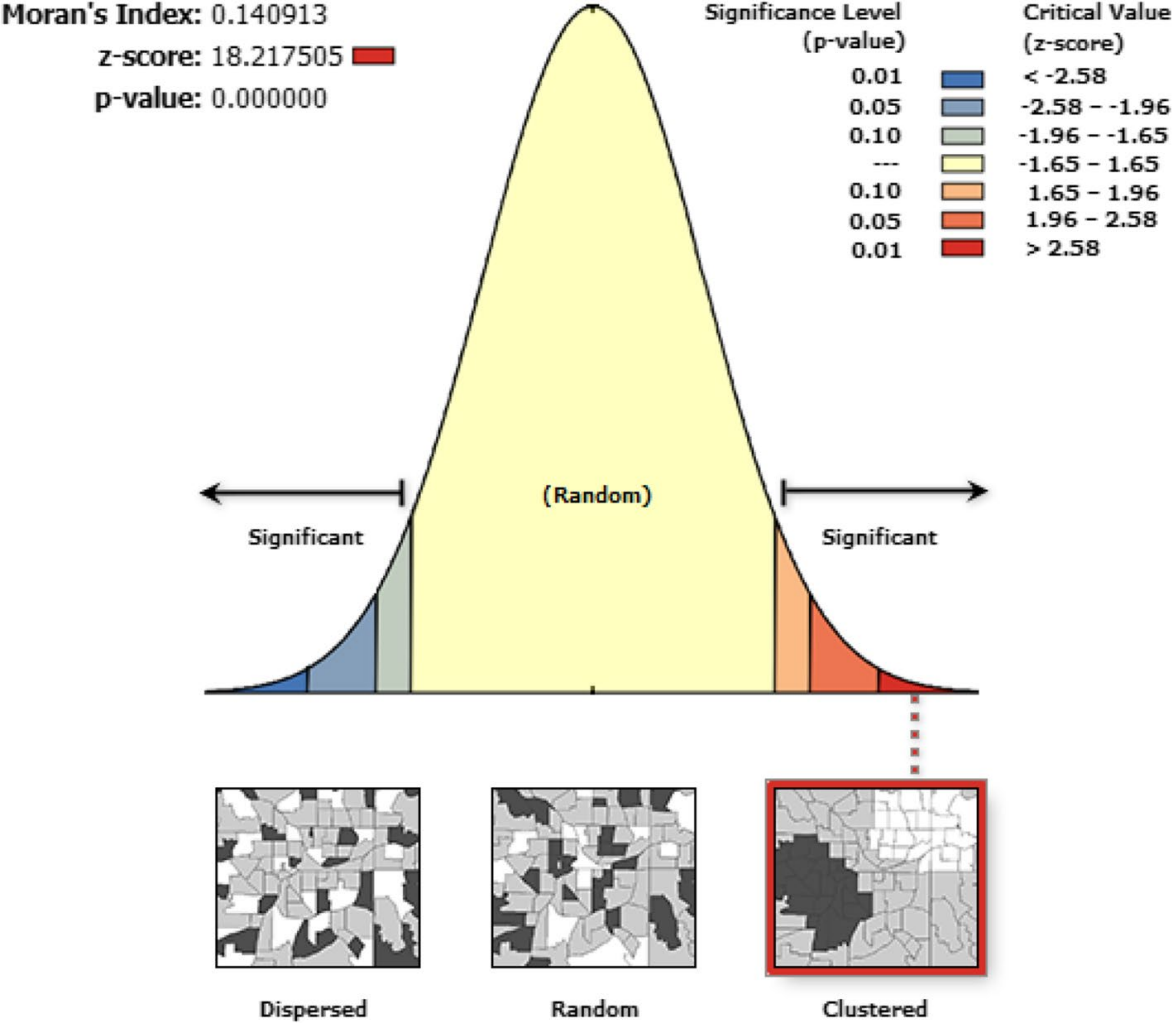
Spatial autocorrelation of childhood anemia in Ethiopia, 2016

The analysis of spatial autocorrelation indicated that the spatial distribution of childhood anemia was non-random in Ethiopia. The Global Moran’s I value 0.14 (*p*-value < 0.0001) indicated that there was significant clustering of childhood anemia in the study area (Fig. 4).

### Hot and cold spot analysis of childhood anemia in Ethiopia

**Fig. 5:**
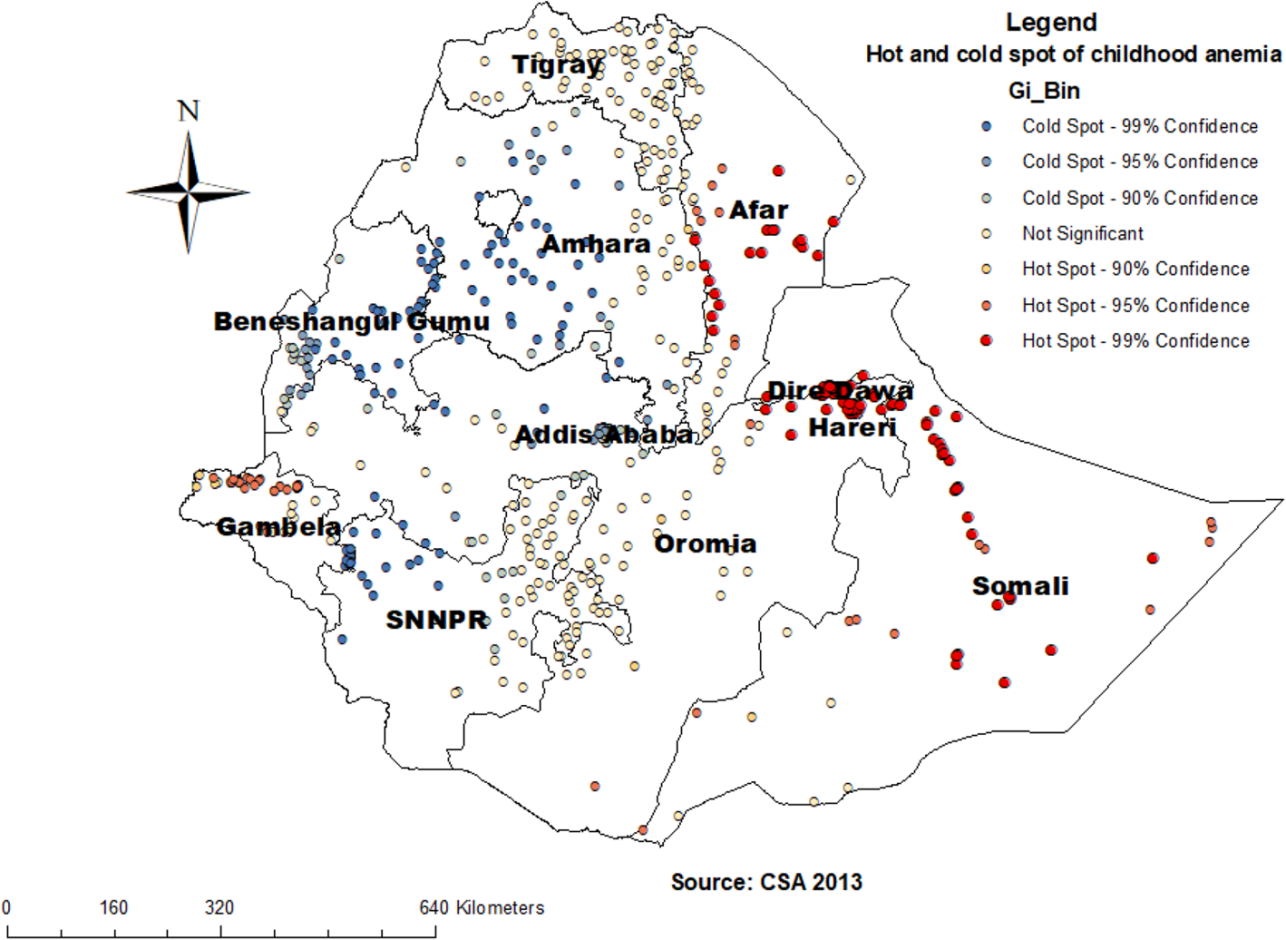
Hot and cold spot analysis of childhood anemia in Ethiopia, 2016

In the Getis OrdGi statistical analysis, significant hotspot areas (high risk of childhood anemia) were aggregated in North and south Eastern part of Somali, Northwest and Sothern part of Afar, Northern part of Gambella, Dire Dawa, and Hareri, while the cold spot areas (low-risk childhood anemia) were found southwest Amhara, Addis Ababa, North West SNNPR and North and South part of Beneshangul Gumuz (Fig. 5).

### The spatial interpolation analysis result

**Fig. 6:**
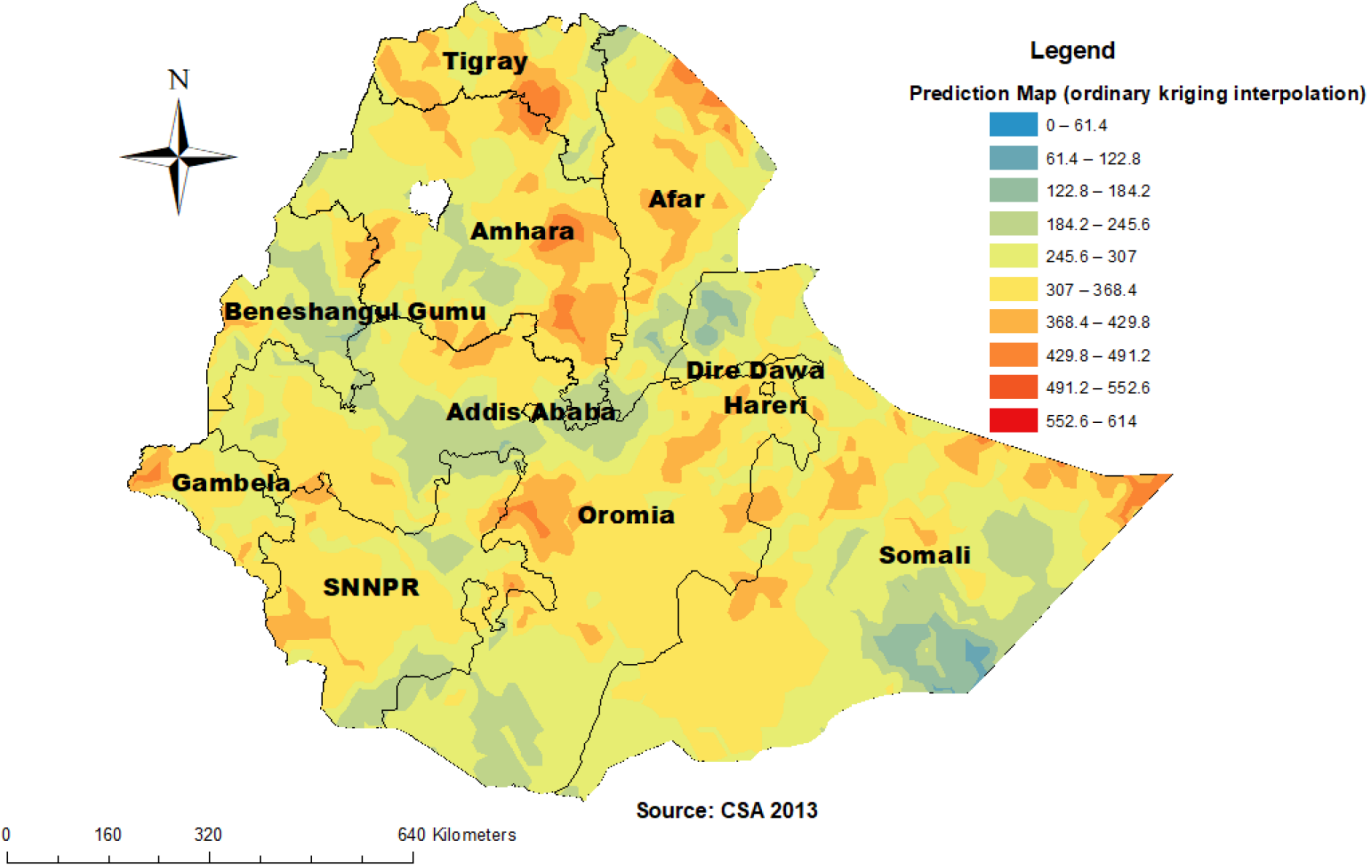
Spatial interpolation of childhood anemia in Ethiopia, 2016

The spatial ordinary kriging interpolation analysis predicted high-risk regions for childhood anemia. Predication of high-risk areas was indicated by red predictions. West and eastern part of Somali, the eastern part of Amhara, South East Tigray, Eastern and Southern afar, Harari, and Dire Dawa regions were predicted as more risky areas compared to other regions. To the opposite of this North West Amhara, Addis Ababa, South West Oromia, and Benshangul Gumuz regions were predicated as having the least risk for anemia (Fig. 6).

### Spatial SaT scan analysis of childhood anemia (Bernoulli based model)

**Fig. 7:**
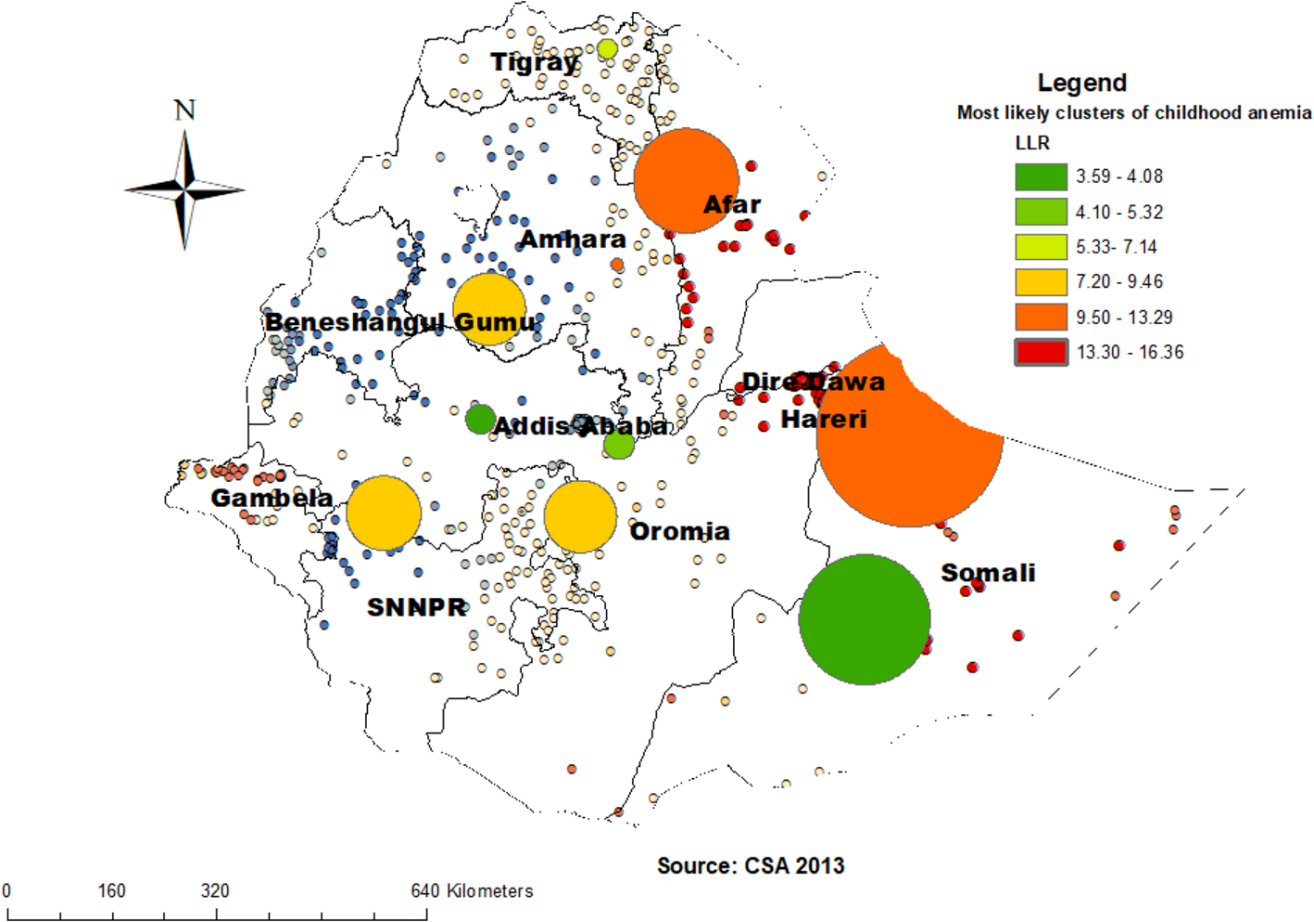
Spatial SaT Scan analysis of childhood anemia in Ethiopia, 2016

A total of 6 clusters with 70 significant enumeration areas were identified and there are two most likely primary clusters and 4 secondary clusters. The primary clusters spatial window encompasses North West of part Oromia, Harari, Dire Dawa, North West Somali, and entire and North West Afar regions. It was centered at .775278 N, 37.939392 E) / 0 km radius and 11.195383 N, 39.269363 E) / 9.21 km, with a relative risk (RR) of 1.67 and Log-Likelihood ratio (LLR) of 16.36 and 13.29, at *p*-value < 0.001. The RR of 1.67 for clusters spatial window means children within the spatial window had a 67% increased risk of anemia than children outside the window (Fig. 7) (Table 2).

**Table 2:** Sat Scan analysis of significant clusters of childhood anemia in Ethiopia among children aged 6 to 59 months, 2016

Clusters	Enumeration areas (clusters) detected	Coordinates/radius	Population	Cases	RR	LLR	*P*-value
1 (1)	301	(7.775278 N, 37.939392 E) / 0 km	32	32	1.67	16.36	< 0.001
2 (2)	421, 8	(11.195383 N, 39.269363 E) / 9.21 km	26	26	1.67	13.29	< 0.001
3 (41)	202, 117, 316, 620, 328, 294, 616, 351, 127, 579, 516, 601, 634, 573,156, 5, 15, 290, 104, 268, 551, 613, 497, 85, 292, 512, 555, 16, 566, 539, 276, 392, 424, 481, 633, 627, 137, 585, 317, 361, 480		503	354	1.19	12.42	0.001
4 (1)	610	10.102213 N, 34.826457 E) / 0 km	29	28	1.61	10.87	0.008
5 (17)	594, 644, 412, 401, 416, 326, 543, 318, 454, 513, 501, 419, 186, 270,281, 213, 619	12.349981 N, 40.242399 E) / 80.40 km	233	173	1.25	10.68	0.01
6 (8)	574, 499, 635, 82, 95, 11, 146, 625	(7.708677 N, 38.758496 E) / 55.60 km	87	71	1.36	9.46	0.03

### Factors associated with childhood anemia

The null model, variance component analysis was performed to decompose the total variance of childhood anemia. The cluster-level variance which shows the total variance of childhood anemia that can be attributed to the context of the community (cluster) in which the child was living was estimated. The applicability of the multi-level mixed-effects logistic regression model in the analysis was justified by the significance of the community-level variance [community variance = 1.24; standard error (SE) =0.089; *P*-value = 0.001], indicating the existence of significant differences between communities (clusters) regarding the childhood anemia. This was supported by the ICC value (ICC = 0.12) which revealed that 12% of the total variance of childhood anemia in Ethiopia can be attributed to the context of the communities. Since it was above the cut of point.

According to mixed-effects logistic regression analysis age of child, religion, wealth index, mother’s current working status, maternal anemic status, number of under-five children in the household, fever in the last 2 weeks, and stunting remained statistically significant variables for anemia in children aged 6 to 59 months.

The odds of developing anemia in children whose age was between 12 and 23, 24–35, 36–47 and 48–59 months is lower by 32% (AOR =0.68, 95%CI = 0.55–0.85), 62% (AOR = 0.38, 95%CI = 0.31–0.47), 75% (AOR = 0.25, 95%CI = 0.2–0.31) and 84% (AOR = 0.16, 95%CI = 0.13–0.20) respectively as compared with those whose age was between 6 and 11 months.

The odds of developing anemia in children having Muslim mother is 2.07 times (AOR = 2.07, 95%CI = 1.75–2.46) that of children having Orthodox mother. Regarding the wealth index, The odds of developing anemia in children with family wealth index of poor, middle, richer and richest is lower by 26% (AOR =0.74, 95%CI = 0.63–0.88), 40% (AOR =0.60, 95%CI = 0.50–0.72), 35% (AOR = 0.65, 95%CI = 0.53–0.79) and 43% (AOR = 0.57, 95%CI = 0.43–0.74) as compared with those with family wealth index of poorest.

Children whose mother was currently working are lower by 13% (AOR =0.87(95%CI = 0.76–0.99) developing childhood anemia than their counterparts. Regarding maternal anemia status, children whose mother was anemic is 1.53 times (AOR =1.53, 95%CI = 1.35–1.73) more likely to develop anemia as compared to children whose mother is free from an anemic.

The odds of developing anemia in children whose household had three or more under-five children is 1.19 times (AOR = 1.19, 95%CI = 1.03–1.38) that of children whose household had one or two under-five children.

Children who had a fever within the last 2 weeks preceding the interview are 1.36 times (AOR =1.36, 95%CI = 1.13–1.65) more likely to develop childhood anemia as compared to those who had no fever within the last 2 weeks.

The odds of developing anemia in a child who had moderate and severe stunting is 1.30 (AOR = 1.30, 95%CI = 1.13–1.50) and 1.82 (AOR = 1.82, 95%CI = 1.54–2.16) times that of those who had no stunting (Table 3).

**Table 3:** i-variable and multi-variable mixed-effects logistic regression analysis for determinants of anemia among children aged 6 to 59 months in Ethiopia from January 18 to June 27 in 2016 (*N* = 7794)

Variables	Anemic status		COR (95%)	AOR (95%CI)
	Non-anemic	Anemic		
**Residency**				
urban	608	719	1	1
rural	2496	3971	1.47 (1.20–1.80)	0.89 (0.68–1.17)
**Age of child in months**				
6–11	200	714	1	1
12–23	497	1268	0.71 (0.57–0.86)	0.68 (0.55–0.85)*
24–35	646	1056	0.40 (0.33–0.49)	0.38 (0.31–0.47)*
36–47	770	886	0.26 (0.21–0.32)	0.25 (0.20–0.31)*
48–59	991	766	0.17 (0.14–0.21)	0.16 (0.13–1.20)
**Maternal educational level**				
No education	1903	3181	1	1
primary	840	1141	0.90 (0.79–1.02)	0.95 (0.82–1.11)
secondary	233	253	0.75 (0.60–0.93)	0.83 (0.63–1.10)
higher	128	115	0.60 (0.44–0.82)	0.76 (0.51–1.12)
**Religion of mother**				
orthodox	1243	1107	1	1
Protestant	641	776	1.24 (1.02–1.49)	1.213 (0.99–1.48)
Muslim	1145	2692	2.48 (2.11–2.91)	2.07 (1.75–2.46)**
others	75	115	1.71 (1.17–2.49)	1.49 (1.01–2.22)*
**Wealth index**				
poorest	849	2005	1	1
poorer	568	819	0.69 (0.59–0.81)	0.74 (0.63–0.88)*
middle	543	613	0.56 (0.47–0.67)	0.60 (0.50–0.72)*
richer	465	518	0.56 (0.47–0.68)	0.65 (0.53–0.79)*
richest	679	735	0.46 (0.38–0.55)	0.57 (0.44–0.74)*
**Husband’s educational level**				
No education	1317	2305	1	1
primary	1034	1420	0.89 (0.78–1.00)	1.01 (0.88–1.16)
secondary	304	404	0.86 (0.71–1.05)	1.02 (0.81–1.27)
higher	220	294	0.84 (0.67–1.06)	1.26 (0.95–1.68)
**Mother’s current working status**				
Not working	1317	3501	1	1
Working	1022	1187	0.76 (0.68–0.86)	0.87 (0.76–0.99)*
**Maternal anemic status**				
Non-anemic	2291	2708	1	1
Anemic	813	1982	1.60 (1.43–1.80)	1.53 (1.35–1.73)*
**Size of a child at birth**				
Average	1372	1943	1	1
Above average	997	1375	0.95 (0.84–1.07)	0.97 (0.85–1.10)
Below average	700	1338	1.21 (1.07–1.38)	1.11 (0.96–1.27)
**Number of under-five children**				
1–2 children	2622	3655	1	1
≥ 3 children	482	1035	1.27 (1.10–1.46)	1.19 (1.03–1.38)*
**Maternal age in years**				
15–24	620	1137	1	1
25–29	912	1443	0.87 (0.75–1.01)	1.05 (0.89–1.23)
30–34	714	1064	0.83 (0.71–0.96)	1.06 (0.89–1.26)
35–48	858	1064	0.71 (0.61–0.82)	0.95 (0.80–1.13)
**Had diarrhea in the last two weeks**				
No	2762	4079	1	1
Yes	339	604	1.35 (1.15–1.58)	0.90 (0.75–1.07)
Had fever in the last two weeks				
No	2718	3924	1	1
Yes	382	765	1.54 (1.33–1.79)	**1**.36 (1.13–1.65)*
Had to cough in the last two weeks				
No	2615	3853	1	1
Yes	489	836	1.29 (1.12–1.48)	1.02 (0.86–1.21)
**Taking of Vitamin A in the last 6 months**				
No	1445	2482	1	1
Yes	1606	2134	0.86 (0.77–0.95)	0.92 (0.82–1.04)
Taking iron pills, sprinkles or syrup				
No	2786	4288	1	1
Yes	280	350	0.86 (0.71–1.03)	0.99 (0.81–1.22)
**Drugs for intestinal parasites in the last 6 months**				
No	2560	4072	1	1
yes	483	557	0.81 (0.69–0.94)	1.12 (0.95–1.33)
**Stunting status**				
No stunting	2101	2839	1	1
Moderate stunting	588	949	1.24 (1.09–1.41)	1.31 (1.13–1.50)*
Severe stunting	347	751	1.61 (1.38–1.89)	1.82 (1.54–2.16)*
**wasting status**				
No wasting	2793	3980	1	1
Moderate wasting	237	523	1.37 (1.14–1.64)	1.09 (0.89–1.32)
Severe wasting	32	95	1.93 (1.24–3.00)	1.44 (0.91–2.28)
**Random component**				
Community variance			–	1.24 (0.089)
Intra-class correlation coefficient (ICC)				0.12(0.08,0.21)

*p-value < 0.05 statistically significant

## Discussion

The purpose of this study is to investigate spatial variation and determinants of childhood anemia aged 6 to 59 months in Ethiopia by using 2016 EDHS data. The spatial variation of childhood anemia was non-random across the country with Global Moran’s I 0.65 (*p*-value < 0.0001). This indicated that there was significant clustering of childhood anemia in Ethiopia. The Purely Spatial Sat Scan analysis identified primary and secondary significant clusters of childhood anemia.

Different methods of spatial analysis most consistently demonstrate anemia high risk and low-risk regions. The hotspot areas (high risk of childhood anemia) were aggregated in the North and southeastern part of Somali, North West and Sothern part of Afar, Northern part of Gambella, Dire Dawa, and Hareri. Whereas the most significant clusters were North West of part Oromia, Harari, and Dire Dawa, North West Somali, and entire and North West Afar regions. Regional differences in feeding habits, the distribution of infectious diseases, and the availability and access to use maternal and child health care services could be the most important reasons for the result of regional variability of childhood anemia in our country [33]. Geographic variation is the risk of anemia and dynamics of the soil content of minerals since the identified high-risk regions were categorized to Eastern and South-Eastern parts of the country. Meaning high-risk regions share similar environmental conditions as evidenced by boundary formation to each other. In addition, epidemiological factors such as fever and stunting which are identified as determinants of anemia in different studies including our studies were more common in some regions. Similarly, the finding from Nigeria showed a distinct North-South divide in the Hemoglobin concentration of the children and that states in the Northern part possess a higher risk of anemia [11]. Furthermore, our finding is also supported by the study conducted in sub-Saharan Africa which concludes that the distribution of anemia is driven by large-scale environmental factors [8]. These all imply that the dynamics of the mineral content of the soil is the most probable explanation for observed geographical variation in the risk of anemia.

According to mixed-effect logistic regression, age of the child, the religion of mother, wealth index, mother’s current working status, maternal anemic status, number of under-five children in the household, fever in the last 2 weeks, and stunting were statistically significant factors childhood anemia in Ethiopia.

Age is one of the most important predictors of childhood anemia. The odds of developing childhood anemia in children whose age was between 12 and 23, 24–35, 36–47 and 48–59 months is lower by 32, 62, 75, and 84% respectively as compared with those whose age was between 6 and 11 months. This finding is consistent with several studies around the world [9, 10, 12–17, 19–23]. This is due to a wide gap between high iron demand for fast growth [4] and low iron supply because of inappropriate initiation of complementary feeding [12, 34] and the highest depletion of the prenatal iron store starting at 6 months of age [35]. Furthermore, children’s age more than 12 months commonly consume a variety of foods that contain iron contents like meat, fish, poultry, and cereals [36]. In addition, different infectious diseases like intestinal helminths are very common in younger children because they might ingest contaminated things into their mouths compared to older ones, particularly children living in an unsanitary environment [37, 38]. All these reasons in turn younger age which is less than 12 months more prone to anemia.

Wealth index was a determinant factor of childhood anemia in Ethiopia The odds of developing anemia in a child with family wealth index of poor, middle, richer, and richest was lower by 25.8, 40.4, 35.2, and 43.3% compared with those with family wealth index of poorest, respectively. The finding is supported by studies conducted in Brazil [9, 13], Nigeria [11], and Ethiopia [10, 12]. Evidence suggested that children from lower socioeconomic status are susceptible to various nutritional disorders like anemia, as well as prone to easily preventable diseases [39–41]. That is why children from the poorest families are less likely to provide a balanced diet because no capability to afford and utilized diversified foods and they are prone to poor health conditions that cause anemia like parasitic infections [4, 42].

The odds of developing anemia in a child whose mother was currently working is lower by 13.3% as compared with those whose mother was currently not working. This finding was consistent with the previous study conducting Ethiopia [16]. The possible reason could be increased empowerment of working mothers to care for their children and other health-related actions [43, 44]. Therefore, working mothers are exposed to different health education regarding child caring and they know the impact of disease very well.

Maternal anemia is also another important factor of childhood anemia. The odds of having anemia in children whose mother was anemic is 1.53 times as compared to their counterparts. The result is in line with findings in Cuba [19], Burma [13], and Ethiopia [16]. This is the fact that maternal anemia can be a risk factor for the development of childhood anemia [45]. It is explained by the influence of poor maternal iron reserve during pregnancy and breastfeeding on the iron store of their child [46, 47]. Besides, maternal anemia increases the risk of maternal death, preterm birth, and low birth weight [48, 49]. This leads to children being more prone to develop anemia.

The odds of developing anemia in children whose household had three or more under-five children is 1.19 times that of children whose household had one or two under-five children. This result was concordant with previous studies conducted in Brazil [14], Lao People’s Democratic Republic [15], and Ethiopia [16]. The possible reason could be when the number of under-five children increases has an impact on health problems due to competition for foods, getting appropriate health care services this also leads to infections and cross contaminations [50]. This might worsen the quality of care for children, and increase the risk of anemia.

Fever is also one of the determinants of childhood anemia. The odds of developing anemia in children who had a fever within the last 2 weeks preceding the interview is 1.36 times that of children who had no fever within the last 2 weeks. This finding is consistent with studies conducted in Burma [13], Nigeria [11], and Southern Ethiopia [18]. This could be a fever in children that can be caused by different infectious diseases like malaria, septicemia, tuberculosis, and kalazar that cause anemia by infecting and destructing red blood cells or by different mechanisms [51].

Children with moderate or severe stunting were 1.30 and 1.82 times more likely to have anemia than children who were not stunted. This result is in agreement with previous studies conducted in Burma [13], Kenya [20], and Ethiopia [10, 12, 16–18, 24]. Pathophysiological explanation implies reverse causation between stunting and anemia. In malnourished children, gastrointestinal epithelium disturbance leads to the development of anemia by impairing the absorption of nutrients [51]. on the other hand, anemia during the period of rapid growth leads to irreversible growth retardation [4, 46], coexist with other micronutrient deficiencies, and stunting may increase the development of anemia by a synergism association. The problem of anemia continues to be a public health challenge in these countries, particularly in Africa, with serious consequences for the most vulnerable populations such as children and pregnant women. Therefore, to reduce childhood anemia in different settings policies and programs for anemia control should account for the spatial heterogeneity of anemia.

### Strength and limitations of the study

The strengths of this study, first, the study was based on nationally representative weighted data and can be generalized at the national level. Second, the use of spatial analysis to explore the spatial variation and significant hotspot areas of childhood anemia and using the mixed-effect advanced model to get a real estimate. The limitation of this study is some variables there is retrospective nature so mothers were asked to remember events that happen in the past which leads to recall bias and it is secondary data collected at appoint in time so doesn’t show temporal relationship.

## Conclusion

This study found that childhood anemia was not random in Ethiopia. High-risk areas for childhood anemia were found in West of part Oromia, Harari, Dire Dawa, North West Somali, and entire and North West Afar regions of the country. Anemia is a major public health problem in Ethiopia in under-five children. In general, age of child, wealth index, mother’s current working status, maternal anemic status, number of under-five children in the household, fever in the last 2 weeks, and stunting were variables that had achieved statically significant association for childhood anemia. Therefore, high-risk areas for childhood anemia needs to be given priority and targeted with interventions to reduce the burden of childhood anemia in under-five children.

## Data Availability

All relevant data are publically available and here is the link to the Ethiopian Demographic and Health Survey 2016 data and you can access the data by clicking the following link https://dhsprogram.com/data/dataset/Ethiopia_Standard-DHS_2016.cfm?flag=0

## Abbreviations

AIC: Akaikie Information Critreia
AOR: Adjusted Odds Ratio
BIC: Bayesian Information Criteria
COR: Crude Odds Ratio
EDHS: Ethiopian Demographic and Health Survey
ICC: Intra Class Correlation
LLR: Log Likelihood Ratio test
